# Aberrant methylation of *MUC1* and *MUC4* promoters are potential prognostic biomarkers for pancreatic ductal adenocarcinomas

**DOI:** 10.18632/oncotarget.9924

**Published:** 2016-06-08

**Authors:** Seiya Yokoyama, Michiyo Higashi, Sho Kitamoto, Monika Oeldorf, Uwe Knippschild, Marko Kornmann, Kosei Maemura, Hiroshi Kurahara, Edwin Wiest, Tomofumi Hamada, Ikumi Kitazono, Yuko Goto, Takashi Tasaki, Tsubasa Hiraki, Kazuhito Hatanaka, Yuko Mataki, Hiroki Taguchi, Shinichi Hashimoto, Surinder K. Batra, Akihide Tanimoto, Suguru Yonezawa, Michael A. Hollingsworth

**Affiliations:** ^1^ Department of Pathology, Research Field in Medicine and Health Sciences, Medical and Dental Sciences Area, Research and Education Assembly, Kagoshima University, Kagoshima, Japan; ^2^ Center for the Research of Advanced Diagnosis and Therapy of Cancer, Graduate School of Medical and Dental Sciences, Kagoshima University, Kagoshima, Japan; ^3^ Eppley Institute for Research in Cancer, Fred and Pamela Buffet Cancer Center, University of Nebraska Medical Center, Omaha, NE, USA; ^4^ Department of General and Visceral Surgery, University of Ulm, Ulm, Germany; ^5^ Department of Digestive Surgery, Breast and Thyroid Surgery, Graduate School of Medical Sciences, Kagoshima University, Kagoshima, Japan; ^6^ Department of Oral Surgery, Kagoshima University Medical and Dental Hospital, Kagoshima, Japan; ^7^ Department of Digestive and Life-Style Related Diseases, Human and Environmental Sciences, Health Research, Kagoshima University Graduate School of Medical and Dental Sciences, Kagoshima, Japan; ^8^ Department of Biochemistry and Molecular Biology, Eppley Institute for Research in Cancer and Allied Diseases, University of Nebraska Medical Center, Omaha, NE, USA

**Keywords:** pancreas, prognosis, DNA methylation, mucin, PDAC

## Abstract

Pancreatic cancer is still a disease of high mortality despite availability of diagnostic techniques. Mucins (MUC) play crucial roles in carcinogenesis and tumor invasion in pancreatic neoplasms. MUC1 and MUC4 are high molecular weight transmembrane mucins. These are overexpressed in many carcinomas, and high expression of these molecules is a risk factor associated with poor prognosis. We evaluated the methylation status of *MUC1* and *MUC4* promoter regions in pancreatic tissue samples from 169 patients with various pancreatic lesions by the methylation specific electrophoresis (MSE) method. These results were compared with expression of *MUC1* and *MUC4*, several DNA methylation/demethylation factors (e.g. ten-eleven translocation or TET, and activation-induced cytidine deaminase or AID) and CAIX (carbonic anhydrase IX, as a hypoxia biomarker). These results were also analyzed with clinicopathological features including time of overall survival of PDAC patients. We show that the DNA methylation status of the promoters of *MUC1* and *MUC4* in pancreatic tissue correlates with the expression of *MUC1* and *MUC4* mRNA. In addition, the expression of several DNA methylation/demethylation factors show a significant correlation with *MUC1* and *MUC4* methylation status. Furthermore, *CAIX* expression significantly correlates with the expression of *MUC1* and *MUC4*. Interestingly, our results indicate that low methylation of *MUC1* and/or *MUC4* promoters correlates with decreased overall survival. This is the first report to show a relationship between *MUC1* and/or *MUC4* methylation status and prognosis. Analysis of epigenetic changes in mucin genes may be of diagnostic utility and one of the prognostic predictors for patients with PDAC.

## INTRODUCTION

Patients with pancreatic ductal adenocarcinoma (PDAC) have a poor clinical outcome, despite improvements in diagnosis and treatment. The overall five year survival rate for all patients with or without pancreatectomy after diagnosis is 13% in Japan [1, 2]. On the other hand, patients with a successful resection of PDAC at an early stage (Stage IA) have a 46% five year survival rate [1, 3]. Most patients with PDAC are diagnosed in the advanced stages because of the anatomical location of the pancreas, lack of specific symptoms, infiltration to the surrounding organs, or distant metastasis even from a small primary tumor less than 2 cm in diameter. Thus, a diagnostic technique for small pancreatic adenocarcinomas without symptoms is urgently needed.

Mucins (MUC) play crucial roles in carcinogenesis and tumor invasion in pancreatic neoplasms. MUC1 and MUC4 are large membrane-bound glycoproteins that are translated as single polypeptides. These mucins undergo intracellular autocatalytic proteolytic cleavage into two subunits that form stable non-covalent heterodimers that are transported to the cell surface. MUC1 contributes to oncogenesis by promoting the loss of epithelial cell polarity, promoting growth and survival pathways, activating receptor tyrosine kinase signaling pathways, and conferring resistance to the stress-induced cell death pathway [4–6]. MUC4 plays an important role in cell proliferation and differentiation of epithelial cells by inducing specific phosphorylation of ErbB2 and enhancing expression of the cyclin dependent kinase inhibitor p27, which inhibits cell cycle progression [7–15].

MUC1 and MUC4 are often overexpressed in epithelial cancers, and our immunohistochemical studies in the pancreas and/or biomolecular studies have shown the following: (1) aberrant expression of MUC1 and MUC4 are associated with invasive proliferation of tumors and a poor outcome for patients [16, 17]; and (2) the expression of *MUC1*/*MUC4* mRNA is regulated by epigenetic mechanisms such as DNA methylation in the promoter region [18–20].

Some have reported that expression of MUC1 and MUC4 increases with increasing pancreatic intraepithelial neoplasia (PanIN) and/or intraductal papillary mucinous neoplasm (IPMN) grade [17, 21–24]. Alteration of methylation patterns has been reported as important in cancer development and progression [25]. Concerning pancreatic cancer, it has been shown that MUC4 promoter hypomethylation increases with progression of disease from PanIN to frank PDAC [26]. However, the significance of alterations in DNA methylation status in the promoters of *MUC1* and *MUC4* at various stages in the development of PDAC is not fully understood. Recently, it was reported that DNA methyltransferases (DNMT) add a methyl group to a cytosine, generating 5-methylcytosine (5mC) [27] and TET and/or AID/APOBEC (apolipoprotein B mRNA-editing enzyme, catalytic polypeptide-like) family members were demethylated by conversion of 5mC to 5-hydroxymethylcytosine (5hmC) and further oxidized products in mammalian genomes (i.e. active DNA demethylation) [28–30]. In addition, it has been reported that hypoxia upregulates the expression of these DNA demethylation enzymes as well as *MUC1* [31, 32].

In this study, to further elucidate the relationship between epigenetic changes in the *MUC1 and MUC4* promoters, and their expression in pancreatic tissue, we analyzed bisulfite treated DNA samples by the MSE method [33, 34]. As no recent study has evaluated the correlation between *MUC1* or *MUC4* methylation status and progression of PDAC, we analyzed *MUC1* and *MUC4* methylation status in stage-matched tissues to study the relationship between *MUC1 and MUC4* promoter methylation and prognosis.

## RESULTS

### Correlation between DNA hypomethylation status and clinicopathological features

In total, 267 pancreas tissue samples (103 neoplastic and 164 non-neoplastic) were collected from 169 patients (including 98 paired samples) (Table 1). Expression levels of DNA methyltransferases (DNMTs) as DNA methylation factors (*DNMT1* and *DNMT3a*), DNA demethylation factors (*TET1*, *TET2*, *TET3* and *AID*), *CAIX* (as a hypoxia biomarker) and mucins (*MUC1* and *MUC4*) in neoplastic and non-neoplastic samples are summarized in Supplementary Table 1. In general, neoplastic regions expressed lower levels of *TET1*, *TET2* and *DNMT1* than non-neoplastic regions (p<0.001 in all three factors). Conversely, the neoplastic regions expressed more *MUC4* and *CAIX* than the non-neoplastic regions (p<0.001 in both factors). The relationship between *MUC1* and *MUC4* promoter methylation status and clinicopathological information of pancreatic lesions was also investigated. As shown in Table 2, analysis of neoplastic samples revealed significant differences in *MUC1* promoter methylation status based on sex, occurrence of distant metastasis (M), and stage as defined by the Union for International Cancer Control (UICC) (p=0.034, p=0.002 and p=0.021, respectively). Statistically significant differences in *MUC4* promoter hypomethylation were found only in UICC stage (p=0.028). However, no statistically significant differences in *MUC1* and/or *MUC4* promoter hypomethylation were observed based on age, primary tumor site (T), or lymph node involvement (N). For non-neoplastic tissues, we found that the level of *MUC1* promoter hypomethylation was associated with sex (p=0.050), while *MUC4* promoter hypomethylation was principally associated with the occurrence of distant metastasis (p=0.045).

**Table 1:** Clinicopathological features

		n (%)
Sex	M	91 (53.8%)
	F	78 (46.2%)
Age	median (SD)	68 (±10.71)
	60>	124 (73.4%)
	60<	45 (26.6%)
TNM		
T	0	23 (13.6%)
	1	20 (11.8%)
	2	17 (10.1%)
	3	96 (56.8%)
	4	4 (2.4%)
	NA	9 (5.3%)
N	0	92 (54.4%)
	1	68 (40.2%)
	NA	9 (5.3%)
M	0	153 (90.5%)
	1	6 (3.6%)
	NA	10 (5.9%)
Stage	non	23 (13.6%)
	IA	18 (10.7%)
	IB	12 (7.1%)
	IIA	36 (21.3%)
	IIB	62 (36.7%)
	III	3 (1.8%)
	IV	6 (3.6%)
	NA	9 (5.3%)

**Table 2:** Comparison between DNA methylation status and clinical information

		Demethylation status of MUC1									
		neoplastic region					non-neoplastic region				
		n (%)	mean±SD			p value	n (%)	mean±SD			p value
Sex	M	53 (51.5%)	54.71	±	15.1	0.034	88 (53.7%)	60.42	±	15.0	0.050*
	F	50 (48.5%)	47.75	±	18.1		76 (46.3%)	54.92	±	20.0	
Age	>60	75 (72.8%)	49.67	±	15.5	0.104	115 (69.7%)	57.07	±	18.1	0.354
	<60	28 (27.2%)	55.67	±	19.8		50 (30.3%)	59.82	±	16.4	
TNM											
T	T1 & T2	7 (8.5%)	48.02	±	26.6	0.703	36 (27.1%)	62.21	±	13.2	0.052*
	T3 & T4	75 (91.5%)	50.75	±	17.3		97 (72.9%)	55.58	±	18.7	
N	negative	47 (50.0%)	51.82	±	17.2	0.619	88 (56.8%)	60.27	±	16.3	0.061
	positive	47 (50.0%)	50.05	±	17.5		67 (43.2%)	55.02	±	18.6	
M	negative	87 (93.5%)	52.77	±	15.6	0.002	149 (96.8%)	58.40	±	17.5	0.172
	positive	6 (6.5%)	31.20	±	19.6		5 (3.2%)	47.49	±	14.7	
Stage	IA & IB	6 (7.3%)	45.79	±	28.4	0.021**	29 (21.8%)	62.28	±	13.2	0.183**
	IIA & IIB	67 (81.7%)	52.85	±	16.2		96 (72.2%)	56.35	±	18.8	
	III & IV	9 (11.0%)	35.86	±	18.0		8 (6.0%)	51.76	±	14.0	
Historogy	por	5 (9.6%)	50.67	±	11.2	0.911	7 (7.7%)	59.53	±	22.8	0.816
	tub	47 (90.4%)	51.45	±	16.5		84 (92.3%)	58.05	±	16.6	

		Demethylation status of MUC4									
		neoplastic region					non-neoplastic region				
		n (%)	mean±SD			p value	n (%)	mean±SD			p value
Sex	M	53 (51.5%)	72.32	±	17.1	0.628	88 (53.7%)	70.31	±	13.9	0.089*
	F	50 (48.5%)	70.61	±	19.0		76 (46.3%)	66.69	±	13.2	
Age	>60	75 (72.8%)	70.96	±	17.5	0.626	115 (69.7%)	68.53	±	13.5	0.887
	<60	28 (27.2%)	72.88	±	19.5		50 (30.3%)	68.85	±	14.1	
TNM											
T	T1 & T2	7 (8.5%)	60.18	±	26.1	0.08	36 (27.1%)	66.34	±	10.6	0.265*
	T3 & T4	75 (91.5%)	72.62	±	16.9		97 (72.9%)	69.29	±	14.4	
N	negative	47 (50.0%)	68.81	±	19.6	0.313	88 (56.8%)	66.75	±	13.7	0.234
	positive	47 (50.0%)	72.57	±	16.7		67 (43.2%)	69.33	±	13.0	
M	negative	87 (93.5%)	71.55	±	17.7	0.077	149 (96.8%)	68.13	±	13.0	0.045
	positive	6 (6.5%)	57.92	±	23.1		5 (3.2%)	55.96	±	19.8	
Stage	IA & IB	6 (7.3%)	57.61	±	27.6	0.028**	29 (21.8%)	66.36	±	11.1	0.369**
	IIA & IIB	67 (81.7%)	73.96	±	16.0		96 (72.2%)	69.48	±	13.5	
	III & IV	9 (11.0%)	62.70	±	20.6		8 (6.0%)	64.31	±	20.5	
Historogy	por	5 (9.6%)	60.93	±	17.8	0.109	7 (7.7%)	59.70	±	9.9	0.034
	tub	47 (90.4%)	73.91	±	18.5		84 (92.3%)	70.42	±	13.7	

*p* value is calculated by equal variance t. test, Unequal variance t. test (*) or ANOVA test (**).

### Relationship between expression of MUC1 or MUC4 and corresponding DNA hypomethylation status in pancreatic tissues

**Figure 1:**
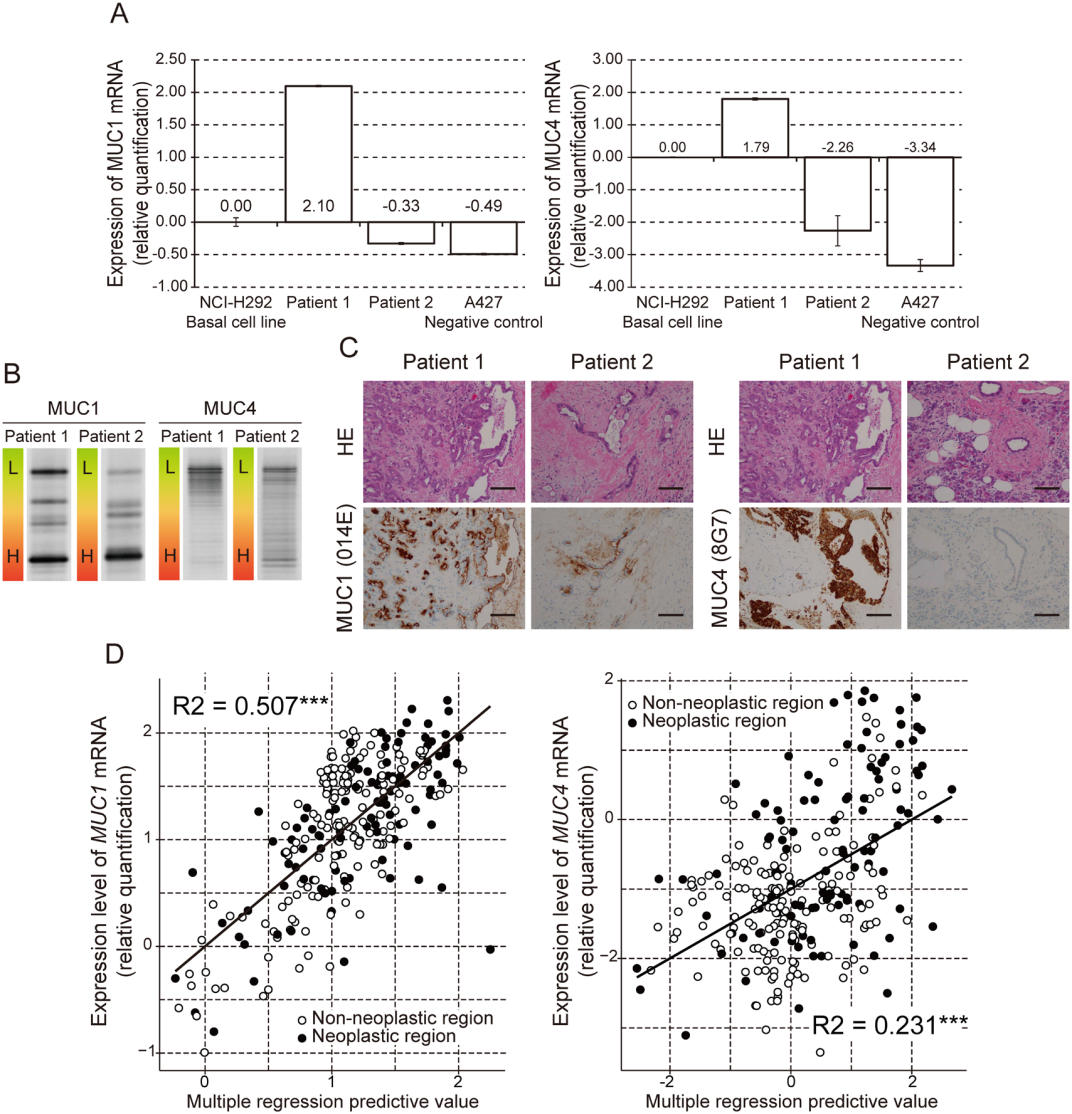
Analysis of MUC1 and MUC4 expression and methylation status in human pancreatic samples **A.** Expression of *MUC1* and *MUC4* mRNA examined by quantitative real time RT-PCR. The bar graphs show gene expression levels relative to those in NCI-H292 cells. The A427 cell line was used as a negative control. **B.** DNA methylation of the *MUC1* and *MUC4* promoter region examined by MSE. L: Low methylated. H: High methylated. Pancreatic tissue from patient 1 showed hypomethylated MUC1 and MUC4. Pancreatic tissue from patient 2 showed hypermethylated MUC1 and MUC4. **C.** Expression of MUC1 and MUC4 protein examined by immunohistochemical staining. HE: Hematoxylin and Eosin Staining. Magnification: ×20. Scale bar: 100 μm. **D.** Multiple regression analysis of mRNA expression against expression level of *CAIX* and DNA hypomethylation status in *MUC1* or *MUC4*. R2: R squared, ***: *p*<0.001, **: *p*<0.01, *: *p*<0.05.

We examined the relationship between mRNA expression, DNA methylation and IHC staining in paired pancreatic tissues. Representative cases of mRNA expression (RT-PCR) paired with IHC analysis and MSE analysis are shown in Figure 1. We found that mRNA positive samples were also IHC positive with corresponding high levels of hypomethylated DNA for both MUC1 and MUC4. On the other hand, mRNA negative samples were IHC negative and showed higher levels of methylated DNA in MUC1 and MUC4 (Figure 1A, 1B and 1C). We analyzed the relationship between hypomethylation status of *MUC1* and *MUC4* and the expression of *MUC1*, *MUC4* and *CAIX* mRNA with Pearson's correlation coefficient (Supplementary Table 2). A good degree of correlation was observed between hypomethylation status and mRNA expression (R=0.436 *p*<0.001 and R=0.317 *p*<0.001, respectively). In addition, a high correlation between expression of *CAIX* and *MUC1* and/or *MUC4* mRNA expression was found (R=0.632 *p*<0.001 and R=0.474 *p*<0.001, respectively). Interestingly, multiple regression analyses showed a closer relationship between *MUC1* mRNA expression and hypomethylation status than *MUC4* mRNA expression and hypomethylation status (R^2^=0.507 *p*<0.001 and R^2^=0.231 *p*<0.001, respectively). The multiple regression predictive value was obtained from following formulas: Fm (expression level of MUC1 mRNA) = (59.8+1.3x(MUC1 hypomethylation index)+30.0x(expression level of CAIX))/100, Fm (expression level of MUC4 mRNA) = −1.288+0.009x(MUC4 hypomethylation index)+0.352x(expression level of CAIX) (Figure 1D). In a pancreatic cancer cell line, hypoxia induced an increase in expression of *MUC4* mRNA (Supplementary Figure 1).

### DNA hypomethylation status and expression of methylation-related enzymes in pancreas tissue

We examined whether the methylation status of mucin genes were influenced by the expression of DNA methylation-related enzymes. In a single regression analysis, the hypomethylation status of *MUC1* showed significant correlation with expression of *TET1*, *TET2*, *AID*, and *DNMT3a* mRNA (R=0.270 *p*<0.001, R=0.202 *p*=0.001, R=−0.288 *p*<0.001 and R=0.348 *p*<0.001, respectively) (Supplementary Table 2). On the other hand, the expression of *TET1*, *TET2* and *DNMT3a* mRNA showed significant correlation with *MUC4* hypomethylation (R=0.280 *p*<0.001, R=0.232 *p*<0.001, and R=0.366 *p*<0.001, respectively) (Supplementary Table 2). The expression of *CAIX* mRNA (as a hypoxia biomarker) showed significant correlation with *TET1*, *TET3*, *DNMT1* and *DNMT3a* mRNA in pancreatic tissue (R=0.262 *p*<0.001, R=0.313 *p*<0.001, R=−0.425 *p*<0.001 and R=0.448 *p*<0.001, respectively) (Supplementary Table 2).

In order to find statistically significant interactions between enzymes related to DNA methylation, we performed a multiple regression analysis. We determined the best regression formula with the least variables (six DNA methylation-related enzymes) and of which the AIC value was lowest for hypomethylation status of MUC1 or MUC4 is as follows: Fm (hypomethylation status of *MUC1*) = 57.102 − 3.789(*TET3*) + 7.553(*DNMT1*) + 24.020(*DNMT3a*) − 8.897(*AID*), Fm (hypomethylation status of *MUC4*)= 57.894 − 3.039(*TET3*) + 17.825(*DNMT3a*).

**Figure 2:**
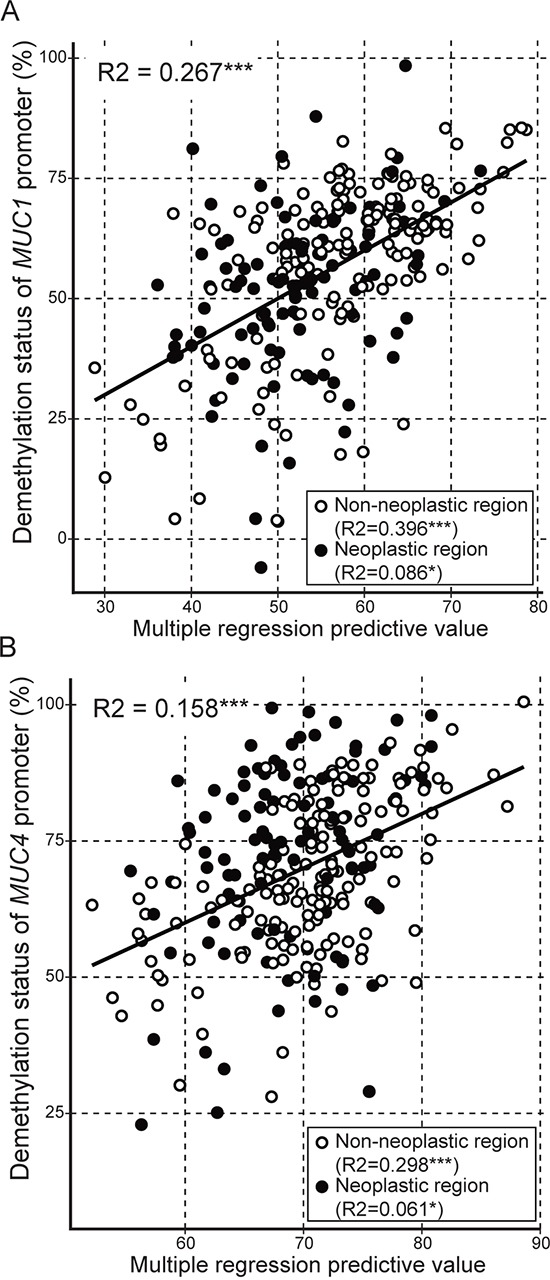
Multiple regression analysis of hypomethylation status of *MUC1* or *MUC4* against expression level of DNA methylation-related enzymes The multiple regression predictive value was obtained from following formulas. **A.** Fm (hypomethylation status of *MUC1*) = 57.102 − 3.789(*TET3*) + 7.553(*DNMT1*) + 24.020(*DNMT3a*) − 8.897(*AID*), **B.** Fm (hypomethylation status of *MUC4*) = 57.894 − 3.039(*TET3*) + 17.825(*DNMT3a*). R2: R squared, ***: *p*<0.001, **: *p*<0.01, *: *p*<0.05, ○: non-neoplastic region, ●: neoplastic region.

Using these models, the observed versus predicted methylation status is shown in Figure 2. The R^2^ values are 0.267 (*p*<0.001) and 0.158, respectively. In the case of *MUC1*, the non-neoplastic sample analysis gave an R^2^ value of 0.369 (*p*<0.001), and the neoplastic sample analysis gave an R^2^ value of 0.086 (*p*=0.021). In the case of *MUC4*, the non-neoplastic sample analysis gave an R^2^ value of 0.298 (*p*<0.001), and the neoplastic sample analysis gave an R^2^ value of 0.061 (*p*=0.016).

### DNA methylation status and PDAC prognosis

**Figure 3:**
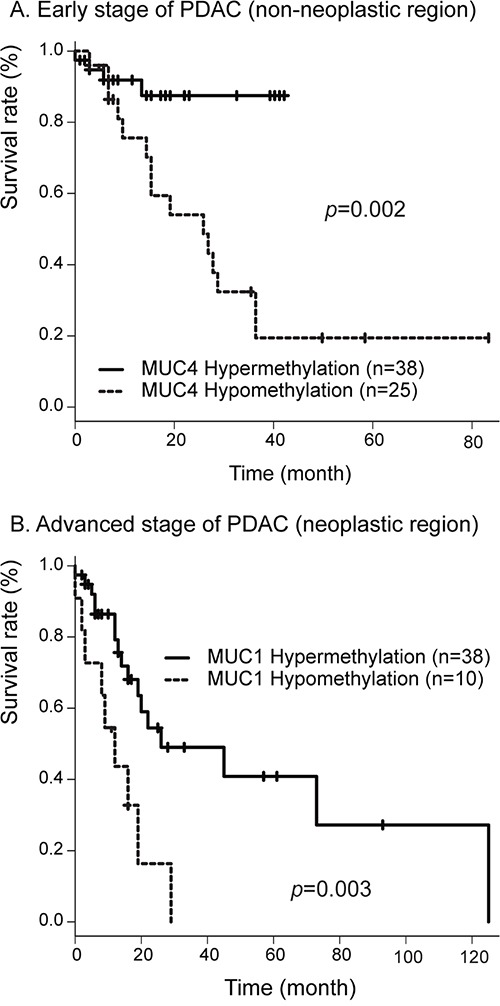
**A.** Correlation between *MUC4* hypomethylation status in non-neoplastic region and overall survival in 66 patients with early stage PDAC determined by the Kaplan-Meier method. **B.** Correlation between *MUC1* hypomethylation status in neoplastic region and overall survival in 71 patients with advanced stage PDAC determined by the Kaplan-Meier method.

To investigate whether the methylation status of *MUC1* and *MUC4* correlated with survival, we compared overall survival between a *MUC1* or *MUC4* hypermethylation group and a *MUC1* or *MUC4* hypomethylation group. In the whole group, we found that patients with *MUC4* hypermethylation showed a much better prognosis than patients with *MUC4* hypomethylation in neoplastic region and/or non-neoplastic region (data not shown). *MUC1* methylation status showed no correlation with survival. To further assess whether the *MUC1* and/or *MUC4* methylation status affect prognosis, we divided patients into two groups based on the presence or absence of lymph node metastasis. In early stages (IA, IB and IIA) of PDAC samples, of the 66 patients (2 neoplasm only and 34 non-neoplasm only), 20 died during the follow-up period (0–125 months). The median overall survival was 30 months. The patient group showing *MUC4* hypermethylation (< 70.58) in non-neoplastic region showed much better prognosis than the group with *MUC4* hypomethylation in non-neoplastic regions (HR=4.78, IC 1.57-14.49, *P*=0.002 by log rank test) (Figure 3). Similarly, the patient group showing *MUC4* hypermethylation (< 72.00) in neoplastic regions showed a much better prognosis than the group showing *MUC4* hypomethylation in neoplastic regions (HR=2.60, IC 0.94-7.19, *P*=0.048 by log rank test). However, hypomethylation status of *MUC1* showed no correlated with survival.

In advanced stages (IIB, III and IV) of PDAC, of the 71 patients (2 neoplasm only and 21 non-neoplasm only), 32 died during the follow-up period (0–125 months). The median overall survival was 22 months. The patient group showing *MUC1* hypermethylation (< 46.87) in neoplastic regions showed better prognosis than the group with MUC1 hypomethylation in neoplastic regions (HR=2.43, IC 1.44-7.89 *P*=0.003 by log rank test) (Figure 3). Similarly, the patient group showing *MUC4* hypermethylation (< 75.58) in neoplastic regions showed better prognosis than the group with *MUC4* hypomethylation in neoplastic regions (HR=2.47, IC 1.10-5.56, *P*=0.024 by log rank test). However, hypomethylation status of *MUC1* or *MUC4* in non-neoplastic region showed no correlation with survival.

**Figure 4:**
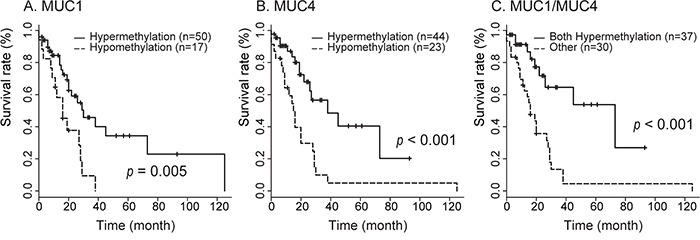
Correlation between *MUC1* and/or *MUC4* hypomethylation in neoplastic region status and overall survival in 98 patients with PDAC in stage IIA and IIB determined by the Kaplan-Meier method Survival of patients with *MUC1* **A.**, *MUC4* **B.**, and *MUC1*/*MUC4* **C.** hypomethylation status was worse than that for hypermethylation.

With respect to stage IIA and IIB PDAC samples, of 98 patients evaluated (2 neoplasm only and 31 non-neoplasm only), 45 died during the follow-up period (0–125 months). The median overall survival was 27 months. Analysis of Kaplan-Meier survival curves showed that patients with hypomethylation of *MUC1* displayed a significant decrease in overall survival as compared to those with hypermethylation (< 62.17) of *MUC1* (HR=2.65, IC 1.3-5.4, *p*=0.005 by log-rank test) (Figure 4). Analysis of Kaplan-Meier survival curves showed that patients with hypomethylation of *MUC4* had a significant decrease in overall survival as compared to the group with hypermethylation (< 82.75) of *MUC4* (HR=3.02, IC 1.54-5.93, *P*<0.001 by log-rank test) (Figure 4). Finally, we analyzed the effect of methylation status of both *MUC1* and *MUC4* on overall survival. Using two selected threshold values (*MUC1*, 62.173 and *MUC4*, 82.747), we found a significant association between methylation and survival (HR=3.59, IC 1.75-7.54, *p*<0.001 by log-rank test) (Figure 4). The threshold values of hypomethylation status, AUC and survival rates are summarized in Table 3.

**Table 3:** Relationship between DNA promoter methylation status and prognosis

			T value	AUC	D/n	p/y	HR	IC95	*p*-value
A. Early stages of PDAC (stage IA, IB and IIA)									
1. MUC1	neoplastic region	Hyper	< 46.87	0.691	3/11	0.53	1.00	ref	
		Hypo			13/21	1.48	1.13	0.32-4.05	0.850
	non-neoplastic region	Hyper	< 64.10	0.605	6/30	1.48	1.00	ref	
		Hypo			13/34	1.85	1.73	0.66-4.57	0.260
2. MUC4	neoplastic region	Hyper	< 82.20	0.777	6/21	1.29	1.00	ref	
		Hypo			10/11	0.72	2.60	0.94-7.19	0.048
	non-neoplastic region	Hyper	< 70.57	0.768	4/39	1.78	1.00	ref	
		Hypo			15/25	1.54	4.78	1.57-14.49	0.002
3. MUC1/MUC4	neoplastic region	other	7/22	1.33	1.00	ref			
		Both Hypo	9/10	0.68	2.12	0.78- 5.72	0.130		
	non-neoplastic region	other	8/48	2.44	1.00	ref			
		Both Hypo	11/16	0.88	4.21	1.69-10.49	<0.001		
B. Advanced stages of PDAC (stage IIB, III and IV)									
1. MUC1	neoplastic region	Hyper	< 61.39	0.627	17/39	2.43	1.00	ref	
		Hypo			9/11	0.34	3.36	1.44- 7.89	0.003
	non-neoplastic region	Hyper	< 35.63	0.586	1/10	0.29	1.00	ref	
		Hypo			29/59	3.28	3.06	0.41-22.74	0.250
2. MUC4	neoplastic region	Hyper	< 75.58	0.704	10/29	1.59	1.00	ref	
		Hypo			16/21	1.19	2.47	1.10-5.56	0.024
	non-neoplastic region	Hyper	< 72.00	0.683	12/41	1.74	1.00	ref	
		Hypo			18/28	1.82	1.48	0.69- 3.15	0.309
3. MUC1/MUC4	neoplastic region	Both Hyper	9/28	1.58	1.00	ref			
		other	17/22	1.19	2.90	1.23- 6.63	0.008		
	non-neoplastic region	Both Hyper	4/8	0.38	1.00	ref			
		other	22/61	2.39	1.01	0.35-3.00	0.149		
C. Stage IIA and IIB									
1. MUC1	neoplastic region	Hyper	< 62.17	0.659	22/50	3.07	1.00	ref	
		Hypo			14/17	0.73	2.65	1.32-5.44	0.005
	non-neoplastic region	Hyper	< 64.78	0.634	21/58	2.40	1.00	ref	
		Hypo			22/38	2.45	1.04	0.61-1.87	0.922
2. MUC4	neoplastic region	Hyper	< 82.75	0.763	15/44	2.57	1.00	ref	
		Hypo			21/23	1.22	3.02	1.54-5.39	<0.001
	non-neoplastic region	Hyper	< 72.00	0.703	15/55	2.18	1.00	ref	
		Hypo			28/41	2.67	1.41	0.74-2.71	0.297
3. MUC1/MUC4	neoplastic region	Both Hyper	10/37	2.21	1.00	ref			
		other	26/30	1.59	3.59	1.71-7.54	<0.001		
	non-neoplastic region	Both Hyper	16/48	1.85	1.00	ref			
		other	27/45	3.01	1.04	0.55-1.96	0.895		

T value: Threshold value, AUC: Area under the curve, D/n: Death/number, p/y: person-years, HR: hazard ratio, Hyper: Hypermethylation, Hypo: Hypomethylation.

## DISCUSSION

PDAC is an aggressive malignancy that carries an extremely poor prognosis due to delayed diagnosis, early metastasis and resistance to most cytotoxic agents [1]. Thus, it is very important to establish new diagnostic, prognostic and therapeutic biomarkers. It has been shown previously that expression of mucin genes (including *MUC1*, *MUC2*, *MUC3*, *MUC4* and *MUC5AC*) is regulated by DNA methylation at promoter regions in cancer cell lines [18–20, 35, 36]. In the present study, we analyzed the relationships among the expression of two mucins, *MUC1* and *MUC4*, their DNA methylation status at promoter regions, and expression of DNA methylation-related enzymes in pancreatic tissue in non-neoplastic and PDAC samples. We also evaluated the association between mucin gene methylation status and survival.

An analysis of the correlation between expression and hypoxic environment revealed that *MUC1* and *MUC4* expression was correlated with a hypoxic environment, as was expression of *CAIX*. This is similar to the results of our previous study in which we showed that a hypoxic environment upregulates MUC1 expression in a pancreatic cancer cell line, and hypoxia-inducible MUC1 contributes to hypoxia-driven angiogenesis through the activation of proangiogenic factors in pancreatic cancer [31]. Interestingly, *MUC4* expression showed a similar result in pancreatic tissue. Also, our results in the pancreatic cancer cell line showed that enforcing a hypoxic environment upregulates expression of *MUC4*. Further studies are needed to clarify the biological significance of this observation, but these results suggest that a hypoxic environment is one factor that explains MUC4 expression in pancreatic tumors. Our analysis of DNA methylation revealed a strong relationship between mRNA expression and DNA hypomethylation for *MUC1* and *MUC4*. This is similar to our previous results with pancreatic cancer cell lines and/or pancreatic tissue [33, 34]. These results suggest that both hypoxia and methylation status play key roles in the regulation of expression of *MUC1* and *MUC4* in pancreatic tissue.

Recently, it was reported that members of the TET (Ten-Eleven Translocation) family and/or AID (activation-induced deaminase)/APOBEC family were demethylated by conversion from 5-methylcytosine (5mC) to 5-hydroxymethylcytosine (5hmC) and further oxidized products in mammalian genomes (i.e. active DNA) [29, 30]. Thus, we evaluated differences in expression of DNA methylation-related enzymes in pancreatic neoplastic regions and non-neoplastic regions. We also calculated the rates of association between mRNA expression of DNA methylation-related enzymes and *MUC1* and *MUC4* hypomethylation status. We found that neoplastic regions showed lower expression of *TET1*, *TET2* and *DNMT1* than non-neoplastic regions. This result suggested that neoplastic regions have altered regulation of epigenetic status. A multiple regression analysis revealed significant correlations for non-neoplastic samples between promoter hypomethylation status and the expression of enzymes related to DNA methylation. However, neoplastic samples showed no correlation between promoter hypomethylation status and expression of enzymes related to DNA methylation. These results suggest that epigenetic regulation of *MUC1* and *MUC4* by these enzymes was ineffective or altered in neoplastic regions.

A previous study showed that analysis of DNA methylation status in promoters of *MUC1*, *MUC2* and *MUC4* (MSE analysis of pancreatic juice samples) could distinguish between gastric type intraductal papillary mucinous neoplasm (IPMN), intestinal type IPMN, other type IPMN and PDAC [33]. In this study, we evaluated the relationship between DNA hypomethylation status and overall survival in PDAC, especially patients in stages IIA and IIB. Those patients with hypomethylated *MUC1* had a significantly decreased overall survival as compared to those with hypermethylated *MUC1*. A similar result was found for *MUC4*. When considered together, the methylation status of *MUC1* and *MUC4* was predictive of survival: patients with hypermethylation of both genes had significantly increased overall survival. Thus, we propose that aberrant methylation of *MUC1* and *MUC4* promoters are potential prognostic biomarkers for PDAC, and suggest that further MSE analysis of human clinical samples to determine its utility for early diagnosis of pancreatic neoplasms and for stratifying patients with respect to modes of treatment.

In summary, our data demonstrate that *MUC1* and *MUC4* expression are increased by hypoxia and DNA hypomethylation. Furthermore, *MUC1* and *MUC4* hypomethylation status is statistically associated with development of distant metastasis, tumor stage and overall survival for PDAC (stage IIA and IIB) patients. Thus, detection of *MUC1* and *MUC4* methylation status has potential prognostic value as an indicator of overall survival and should be evaluated further for clinical utility.

## MATERIALS AND METHODS

### Cell lines

Human pancreatic cancer cell lines BxPC3, HPAF2, Panc1, human colon adenocarcinoma cell lines Caco2, LS174T, and human lung adenocarcinoma cell line A427, NCI-H292 were obtained from the American Type Culture Collection. HPAF2, LS174T, and Caco2 cells were cultured in Eagle's MEM (Sigma, MO, USA), PANC1 and A427 cells were cultured in DMEM (Sigma, MO, USA), and BxPC3 and NCI-H292 cells were cultured in RPMI 1640 (Sigma, MO, USA). The media was supplemented with 10% fetal bovine serum (Invitrogen, Tokyo, Japan) and 100 U/mL of penicillin and 100 μg/mL streptomycin (Sigma, MO, USA). Hypoxic culture conditions were achieved with a multi-gas incubator containing a gas mixture of 94% N_2_, 5% CO_2_ and 1% O_2_ (ASTEC, Fukuoka, Japan).

### Clinical samples

#### Pancreatic tissue samples

We obtained 267 surgically resected fresh tissue blocks (about 2×2×2 mm) with neoplastic or non-neoplastic areas from 169 patients. Table 1 summarizes the clinicopathological features of the 103 neoplastic samples and 164 non-neoplastic samples (including 98 paired samples). 103 patient samples (37 neoplastic samples and 98 non-neoplastic samples) were collected in Kagoshima University from August 2007 to May 2014, and 66 patient samples (66 neoplastic samples and 66 non-neoplastic samples) were collected in Ulm University from February 2001 to February 2013.

### Ethics statement

The study was conducted in accordance with the guiding principles of the Declaration of Helsinki. Collection of samples was approved by the ethical committees of each hospital (Kagoshima University Hospital and Ulm University Hospital), and informed written consent was obtained from each patient. All studies using human materials in this article were approved by the Ethical Committee of Kagoshima University Hospital (revised 20–82, revised 22–127 and revised 26–145).

### Extraction and quantification of mRNA

Total RNA was extracted from cell lines, human pancreatic tissues and pancreatic juices using an RNeasy Mini kit (QIAGEN, Tokyo, Japan). Total RNA (1 μg) was reverse transcribed with a high capacity RNA-to-cDNA Kit (Applied Biosystems, CA, USA). Real-time reverse transcription–PCR was performed on a Roche LightCycler^®^ 96 System using FastStart Essential DNA Green Master (Roche, Tokyo, Japan). Gene expression was normalized to the β-actin mRNA level in each sample. The data normalized were using NCI-H292 cell line. A427 cell line was used as negative control. Primer sets are shown in Supplementary Table 3.

### Extraction of DNA and bisulfite modification

DNA from cell lines, pancreatic tissues, and pancreatic juice was extracted using a DNeasy Tissue System (QIAGEN). Bisulfite modification of the genomic DNA was carried out using an Epitect Bisulfite Kit (QIAGEN). Purification of PCR products was carried out using a Wizard SV Gel and PCR Clean-Up System (Promega KK, Tokyo, Japan).

### MSE analysis

MSE analysis was performed using previously described methods [33, 34]. The target DNA fragments were amplified by nested PCR using bisulfite treated DNA using the primer sets shown in Supplementary Table 3. In the electrophoresis step, the amplicon was applied to the D-Code system (BioRad Laboratories, Hercules, CA, USA) using a polyacrylamide gel with a linear denaturant gradient at 60°C and 70 V for 14 h. Band intensity was quantified by Image J software. The hypomethylation index was calculated as the proportion of highest band intensity/total band intensity of the sample. Subsequently, the hypomethylation index in each sample was normalized using data from a hypomethylated and hypermethylated cell line. Cell lines with hyper- and hypomethylated of *MUC1* (Caco2 and LS174T) and *MUC4* (Caco2 and LS174T) were used as control standards.

### Statistical analysis

Data were analyzed using the “R” computing environment [37]. The normality of the data distribution was evaluated by the Kolmogorov-Smirnov test. F test performed to compare the variances of two samples from normal populations. A non-parametric test of two-group difference was performed by the Mann-Whitney U test. A parametric test of two-group difference was performed by the Welch t-test (Unequal variance) or Student t-test (Equal variance). Bartlett test performed to compare the variances of multi samples from normal populations. A nonparametric test of multi-group difference was performed by the Kruskal-Wallis one-way analysis of variance. A parametric test of multi-group difference was performed by the one-way analysis of variance (ANOVA). The correlation coefficient (R) was determined by the Pearson product-moment correlation coefficient. The multiple regression analysis was performed by general linear model and coefficient of determination (R squared) was determined. Survival rate analysis was evaluated by the Cox proportional hazard model, and threshold points were determined by ROC curve analysis. A *p*-value<0.05 was considered statistically significant.

### Immunohistochemistry

Immunohistochemistry (IHC) was performed in cut sections of pancreatic tumors using anti-MUC1 monoclonal antibody (MAb) clone 014E (MAb MUC1/014E, the kind gift of Suguru Yonezawa) [38] and anti-MUC4 MAb clone 8G7 (MAb MUC4/8G7, the kind gift of Surinder K. Batra) [39] using the immunoperoxidase method. Antigen retrieval was performed using CC1 antigen retrieval buffer (pH 8.5, EDTA, 100°C, 30 minutes; Ventana Medical Systems, AZ, USA) for all sections. Following incubation with the primary antibodies (MAb MUC1/014E diluted 1:5, 37°C, 32 minutes; MAb MUC4/8G7 diluted 1:3000, 37°C, 32 minutes) in phosphate buffered saline, pH 7.4 (PBS) with 1% bovine serum albumin (BSA), sections were stained on a Benchmark XT automated slide stainer using a diaminobenzidine detection kit (UltraView DAB, Ventana Medical Systems). The control staining (normal mouse serum or PBS-BSA instead of the primary antibodies) showed no reaction.

## SUPPLEMENTARY FIGURE AND TABLES


