# Supplementary material for: Aberrant methylation of MUC1 and MUC4 promoters are potential prognostic biomarkers for pancreatic ductal adenocarcinomas
Source: Oncotarget. 2016 Jun 8;7(27):42553–65. doi: 10.18632/oncotarget.9924 (PMC5173155; doi:10.18632/oncotarget.9924)
Supplement: Supplementary file 1 [file oncotarget-07-42553-s001.pdf]

Aberrant methylation of *MUC1* and *MUC4* promoters are potential prognostic biomarkers for pancreatic ductal adenocarcinomas

SUPPLEMENTARY FIGURE AND TABLES

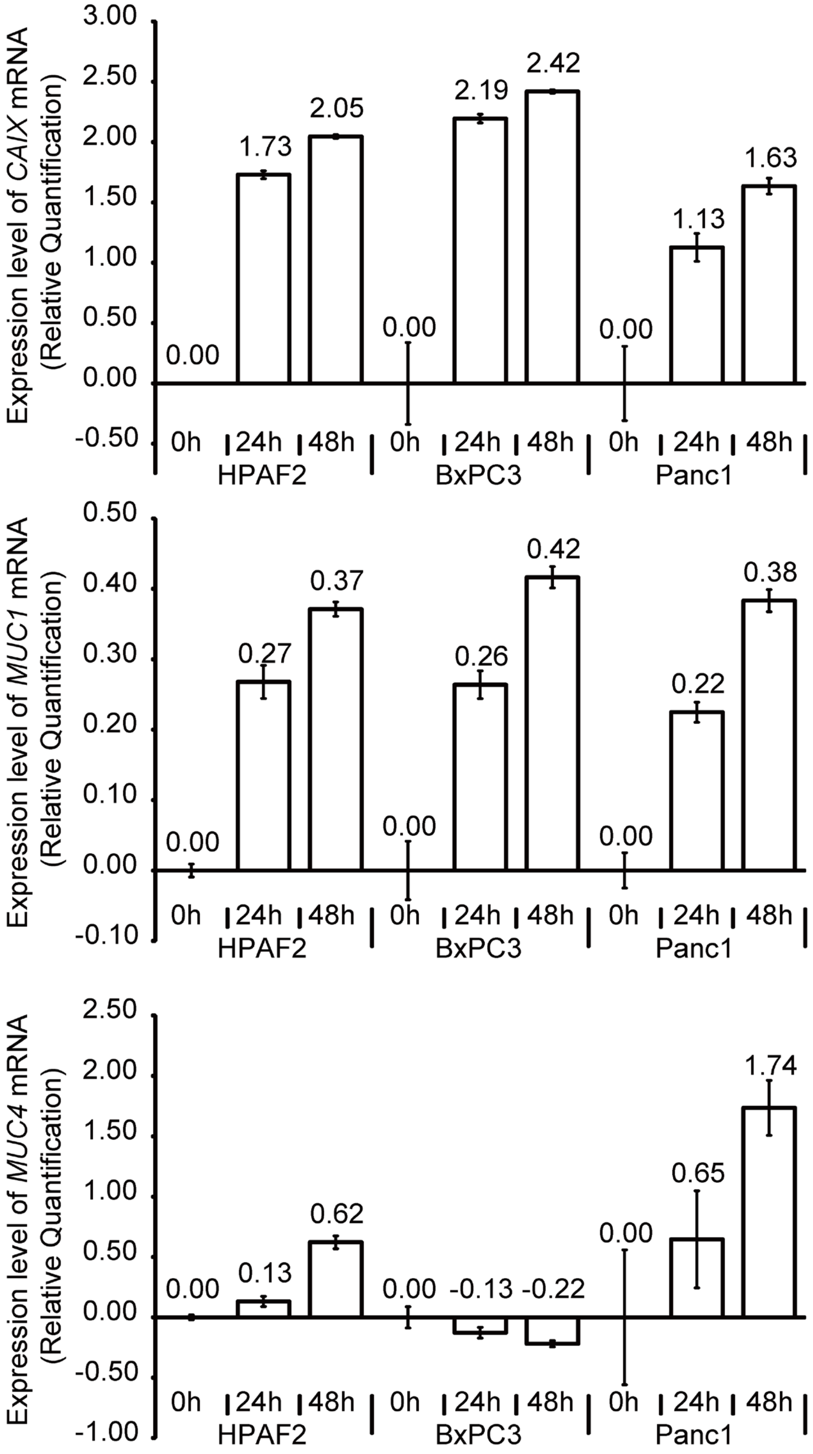

Supplementary Figure S1: Effect of hypoxia treatment for expression level of *MUC1* and *MUC4* mRNA. Expression of *CAIX*, *MUC1* and *MUC4* mRNA examined by quantitative real time RT-PCR. The bar graphs show gene expression levels relative to those in non-treatment of hypoxia.

Supplementary Table S1: Comparison between neoplastic and non-neoplastic regions

	Neoplastic region		Non-neoplastic region		*p* value
	n	mean ± sd	n	mean ± sd	
Methylation status of					
MUC1	103	51.3 ± 17.1	164	58.0 ± 17.6	<0.001**
MUC4	103	71.7 ± 18.1	164	68.6 ± 13.7	0.145*
mRNA expression level of					
MUC1	103	1.2 ± 0.7	163	1.1 ± 0.7	0.443**
MUC4	103	-0.4 ± 1.2	162	-1.2 ± 0.8	<0.001*
TET1	101	0.0 ± 0.5	161	0.2 ± 0.6	<0.001**
TET2	103	0.6 ± 0.5	163	0.8 ± 0.3	<0.001**
TET3	103	0.0 ± 0.4	163	0.0 ± 0.4	0.343**
AID	103	2.0 ± 0.7	163	1.9 ± 0.8	0.294
DNMT1	103	-0.1 ± 0.5	164	0.1 ± 0.4	<0.001
DNMT3a	103	0.6 ± 0.3	163	0.7 ± 0.3	0.059
CAIX	103	-0.1 ± 1.3	162	-0.9 ± 1.1	<0.001*

p value is calculated by equal variant t.test, Unequal variant t.test (*) or Mann-Whitney U test (**).

Supplementary Table S2: Summarized correlation coefficient (R)

		Epression level of mRNA							
		MUC1	***MUC4***	***TET1***	***TET2***	***TET3***	***Dnmt1***	***Dnmt3a***	***AID***
Methylation status									
MUC1	R	0.436	N/A	0.270	0.202	0.010	-0.008	0.348	-0.288
	(*p* value)	(<0.001)		(<0.001)	(0.001)	(0.643)	(0.747)	(<0.001)	(<0.001)
MUC4	R	NA	0.317	0.280	0.232	0.026	-0.081	0.366	0.037
	(*p* value)		(<0.001)	(<0.001)	(<0.001)	(0.263)	(0.083)	(<0.001)	(0.293)
Expression level of mRNA									
MUC1	R		NA	0.430	0.239	0.306	-0.326	0.540	-0.182
	(*p* value)			(<0.001)	(<0.001)	(<0.001)	(<0.001)	(<0.001)	(0.003)
MUC4	R	NA		-0.134	-0.005	-0.048	-0.034	0.038	0.269
	(*p* value)			(0.030)	(0.934)	(0.441)	(0.583)	(0.547)	(<0.001)
CA9	R	0.632	0.474	0.262	0.193	0.313	-0.425	0.448	0.076
	(*p* value)	(<0.001)	(<0.001)	(<0.001)	(<0.001)	(<0.001)	(<0.001)	(<0.001)	(0.447)

Supplementary Table S3: Synthetic oligonucleotides used in the study

**RT-PCR**			
	MUC1		Forward: 5’-CCAGCACCGACTACTACCAAGAG-3’
			Reverse: 5’-CGTCGTGGACATTGATGGT-3’
	MUC4		Forward: 5’-TGGGACGATGCTGACTTCTC-3’
			Reverse: 5’-CCCCGTTGTTTGTCATCTTTC-3’
	TET1		Forward: 5'-CCCGAATCAAGCGGAAGAATA-3'
			Reverse: 5'-TACTTCAGGTTGCACGGT-3'
	TET2		Forward: 5'-AAGGCTGAGGGACGAGAACGA-3'
			Reverse: 5'-TGAGCCCATCTCCTGCTTCCA-3'
	TET3		Forward: 5'-CCTGCCGATGACAAGCTGGA-3'
			Reverse: 5'-GAGTTCCCGGATAGAGGCGA-3'
	AID		Forward: 5'-AAAATGTCCGCTGGGCTAAG-3'
			Reverse: 5'-AGGTCCCAGTCCGAGATGTAG-3'
	DNMT1		Forward: 5'-GAGGAAGCTGCTAAGGACTAGTTC-3'
			Reverse: 5'-ACTCCACAATTTGATCACTAAATC-3'
	DNMT3a		Forward: 5'-ACAAGAATGCCACCAAAGCAG-3'
			Reverse: 5'-TCATCCACCAAGACACAATGC-3'
	CAIX		Forward: 5'-CGGAAGAAAACAGTGCCTATGAG-3'
			Reverse: 5'-CAGGGCGGTGTAGTCAGAGA-3'
	β-actin		Forward: 5'-CTCTTCCAGCCTTCCTTCCTG-3'
			Reverse: 5'-GAAGCATTTGCGGTGGACGAT-3'
MSE			
	MUC1	1st	Forward: 5’-AAAGGGGGAGGTTAGTTGGA-3’
			Reverse: 5’-TACCCCTCACCTATAAACAC-3’
		2nd	Forward: 5’-[GC clamp*] AAGAGGTAGGAGGTAGGGGA-3’
			Reverse: 5’-AAAACAAAACAAATTCAAAC-3’
	MUC4	1st	Forward: 5’-AGAGTAAGGGGTGTATGGGTG-3’
			Reverse: 5’-AACCTACCCCCTTCATAAC-3’
		2nd	Forward: 5’-[GC clamp*] AGGAGAGAAAAGGGTGATTAG-3’
			Reverse: 5’-ACTCCACTACCCAACAACTAC-3’

*GC clamp: 5'-CGCCCGCCGCGCGCGGCGGGCGGGGCGGGGGCACGGGGGG-3'.
